# Proteomic analysis of Red Sea *Conus taeniatus* venom reveals potential biological applications

**DOI:** 10.1590/1678-9199-JVATITD-2021-0023

**Published:** 2021-10-18

**Authors:** Maged M. A. Fouda, Mohammed Abdel-Wahab, Amal Mohammadien, Mousa O. Germoush, Moustafa Sarhan

**Affiliations:** 1 Department of Biology, College of Science, Jouf University, Saudi Arabia.; 2 Zoology Department, Faculty of Science, Al-Azhar University, Assiut, Egypt.; 3 Department of Biology, College of Science, Taeif University, Saudi Arabia.; 4 Zoology Department, Faculty of Science, Zagazig University, Zagazig, Egypt.

**Keywords:** Conus taeniatus, Conopeptides, Conotoxin, HPLC, Mass spectrometry, Cone snail venom

## Abstract

**Background:**

Diverse and unique bioactive neurotoxins known as conopeptides or conotoxins are produced by venomous marine cone snails. Currently, these small and stable molecules are of great importance as research tools and platforms for discovering new drugs and therapeutics. Therefore, the characterization of *Conus* venom is of great significance, especially for poorly studied species.

**Methods:**

In this study, we used bioanalytical techniques to determine the venom profile and emphasize the functional composition of conopeptides in *Conus taeniatus*, a neglected worm-hunting cone snail.

**Results:**

The proteomic analysis revealed that 84.0% of the venom proteins were between 500 and 4,000 Da, and 16.0% were > 4,000 Da. In *C. taeniatus* venom, 234 peptide fragments were identified and classified as conotoxin precursors or non-conotoxin proteins. In this process, 153 conotoxin precursors were identified and matched to 23 conotoxin precursors and hormone superfamilies. Notably, the four conotoxin superfamilies T (22.87%), O1 (17.65%), M (13.1%) and O2 (9.8%) were the most abundant peptides in *C. taeniatus* venom, accounting for 63.40% of the total conotoxin diversity. On the other hand, 48 non-conotoxin proteins were identified in the venom of *C. taeniatus*. Moreover, several possibly biologically active peptide matches were identified, and putative applications of the peptides were assigned.

**Conclusion:**

Our study showed that the composition of the *C. taeniatus*-derived proteome is comparable to that of other *Conus* species and contains an effective mix of toxins, ionic channel inhibitors and antimicrobials. Additionally, it provides a guidepost for identifying novel conopeptides from the venom of *C. taeniatus* and discovering conopeptides of potential pharmaceutical importance.

## Background

Cone snails are venomous marine mollusks of the genus *Conus* that can produce small cysteine-rich peptides called conotoxins or conopeptides. These conopeptides display diverse pharmacological activities for prey capture, self-defense, competition, and other biological purposes [1,2]. According to their prey preference, cone snails are commonly classified into three main groups: vermivore, molluscivore or piscivore [3,4]. Conopeptides can modulate the nervous system of their targets by affecting ion channels [5-7]. Therefore, conopeptides have become a platform for discovering new drugs in these exceptionally potent venoms. Moreover, specific components in *Conus* venoms are used as therapeutics. For example, ω-MVIIA conotoxin is known commercially as ziconotide (Prialt^®^) and is utilized to cure chronic pain [8-12]. Several other conopeptides are being studied for the treatment of neuropathic pain, epilepsy, hypertension and myocardial infarction [13]. In addition to their contribution to neurobiological and therapeutic applications, conotoxins show high diversity. Conopeptides are stable, relatively small, and structurally diverse with various cysteine frameworks and numerous posttranslational modifications (PTMs) [14-16]. To date, over 800 species of cone snails have been described [17]. Assuming that the venom of each species contains 100 distinct peptides, a repertoire of more than 80,000 conopeptides could be obtained. However, currently only a restricted number of conopeptides (~3%) have been characterized [18,19]. Conopeptides are generated from mRNA-encoded conopeptide precursors that possess signal peptides followed by a variable region and a hypervariable mature peptide [20,21]. At present, conotoxins are classified based on three classification methods: (1) peptide precursor identity, (2) cysteine frameworks, and (3) pharmacological targets and activity. Thus far, twelve families of conotoxins have been identified [18,22].

The worm-hunting cone snail *C. taeniatus* is commonly distributed along the Egyptian Red Sea. However, there is no information regarding its venom composition. Thus, a proteomic analysis of *C. taeniatus* venom is of great interest and essential to uncover its various components. In the present study, high-performance liquid chromatography (HPLC) fractionation combined with LC/mass spectrometry (LC-MS) and offline matrix-assisted laser desorption/ionization (MALDI)-time-of-flight (TOF)-MS was used to assess the conopeptide content in the venom of *C. taeniatus*. This integrated approach provides an initial outline of *C. taeniatus* venom constituents and presents information about potential bioactive peptide candidates that may have pharmaceutical importance. To our knowledge, this is the first proteomic analysis of the venom of Red Sea endemic *Conus* species, and therefore, it provides information that complements and enriches the field of cone toxinology. 

## Methods

### Crude venom extraction

**Figure 1.**
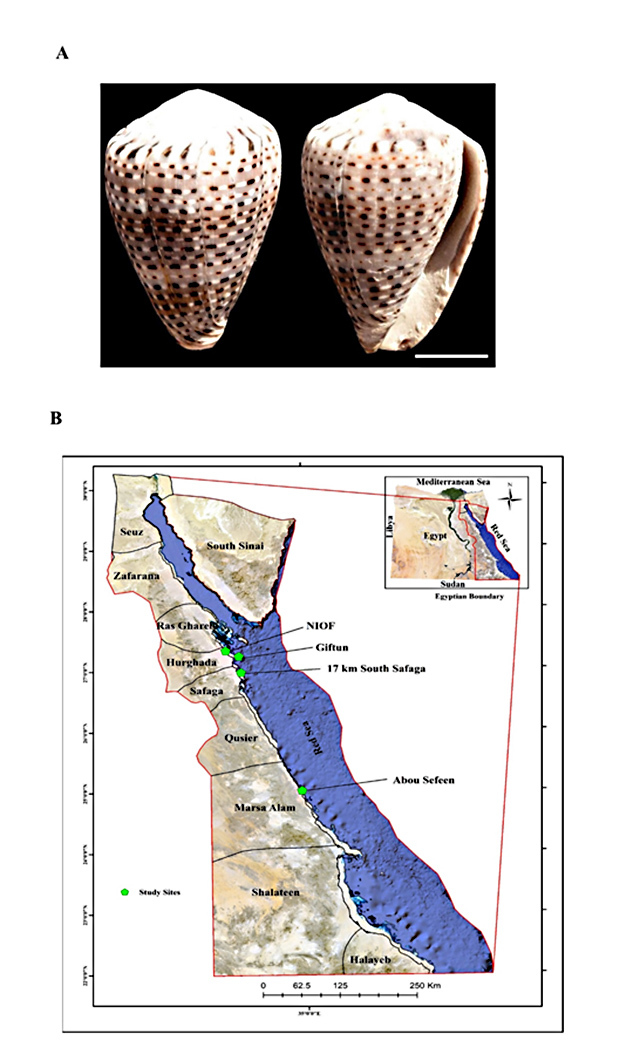
(A) General morphology of *C. taeniatus* shell (bar = 1 cm) and (B) map of the Red Sea in Egypt showing the collection sites of *C. taeniatus*.

Specimens of *C. taeniatus* (n = 40) were collected from several sites along the Red Sea coast of Egypt (Figure 1A and 1B). After carefully dissecting the snail venom apparatus, the venom ducts were sliced into small parts to extract the protein contents. For extraction, parts of the venom ducts were suspended in two percent acetic acid (AA) and then centrifuged at 500 × g for 5 minutes at 4°C. The venom was extracted three times, freeze-dried, and then saved at −80°C until use.

### LC/MS analysis

LC/MS measurements of *C. taeniatus* venom were analyzed using an electrospray ion source (ESI) equipped with an LCMS-IT-TOF (Shimadzu). A reversed-phase C18 HPLC (RP-HPLC) column (Cadenza CD-C18, 2.0 150 mm; Imtakt) was used for separation. The column was eluted with 0.1% formic acid (FA) in H_2_O (solvent A) and 0.1% formic acid in CH3CN (solvent B) at a flow rate of 0.2 mL/min with a linear gradient of 5%-60% solvent B in solvent A, over 55 minutes.

### Reduction and carboxyamidomethylation of the venom

The reduction of crude venom (100 μg) was performed in a buffer containing 0.13 M NaHCO3 (pH 8.5), 2.7 M urea, and 35 mM dithiothreitol (DTT), and then the mixture was incubated at 50°C for one hour under argon gas. The combined reaction mixture was then mixed with iodoacetamide (IAA) at a final concentration of 125 mM and incubated for 1 h at 25°C for the alkylation process. The final mixture including the derivatized peptides was analyzed by LC/MS without purification.

### MALDI-TOF/MS analysis

MALDI-TOF-TOF/MS analysis was performed on a TripleTOF™ 5600+ (AB Sciex, Canada). The venom samples were first desalted by using MonoSpin reversed-phase C18 columns (GlSciences, Cat. No. 5010-21701) prior to the measurement. The venom was dissolved in a matrix solution containing α‐cyano‐4‐hydroxy‐cinnamic acid (HCCA, 2.5 mg, Bruker Daltonics), dissolved in CH3CN (50%, 0.1% formic acid, Sigma‐Aldrich). One µl of the solution was spotted onto a target plate (Bruker Daltonics) and allowed to dry at room temperature. For high precision, external calibration of the sample batches was carried out to correct possible TOF deviation. Measurements were conducted in positive ion mode, and the MS and MS/MS ranges were 400‐1250 and 170‐1500 m/z, respectively. Mass spectra raw files from the TripleTOF^TM^ 5600+ were converted into Mascot generic format (mgf) files using the script provided by AB Sciex and ProteoWizard. The MS/MS spectra were searched using X! Tandem in a Peptide-shaker (v1.16.38) against the UniProt *Conus* organism (Swiss-Prot and TrEMBL containing 10684 proteins) with reversed sequences. With initial mass tolerances of 20.0 and 10.0 ppm, the precursor and fragment masses were established, respectively. The carbamidomethylation of cysteine (mass 57.02 amu) was considered to be a static modification, and the oxidation at methionine (mass 15.99 amu), acetylation of the protein N‐terminus (mass 42.01 amu), deamidation of asparagine (mass 0.98 amu), and deamidation of glutamine (mass 0.98 amu) were considered to be variable modifications. Subsequently, the UniProtKB database (www.uniprot.org) and the Entrez PubMed database (www.ncbi.nih.gov) were used to determine the gene superfamilies found in the crude venom of *C. taeniatus* from known protein fragments.

## Results

### Molecular mass range and distribution of conopeptides detected by LC/MS

**Figure 2.**
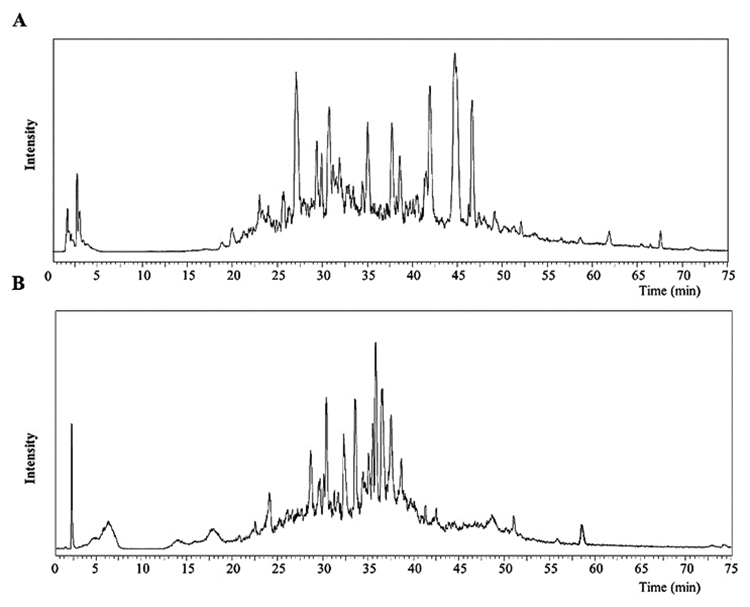
LC/MS chromatograms of **(A)** native and **(B)** Cys-alkylated *C. taeniatus* venom.

**Figure 3.**
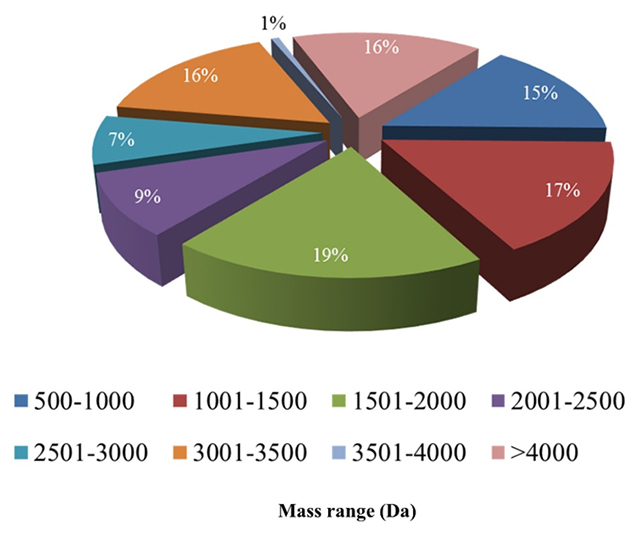
Molecular mass distribution of the components in *C. taeniatus* venom detected by LC/MS.

To study the total number of peptide profiles produced in the venom of *C. taeniatus,* an online LC/MS equipped with an ESI source (LCMS-IT-TOF; Shimadzu) was used to analyze quantified crude venom samples. The LC/MS spectra of the extracted crude venom from *C. taeniatus* demonstrate the remarkable complexity of conopeptides present in this species (Figure 2A and 2B). The LC/MS analysis revealed approximately 149 components from *C. taeniatus* venom. Those between 500 and 4,000 Da represented 84% of the conopeptides, and the large peptides (> 4,000 Da) constituted only 16% of all *C. taeniatus* components (Figure 3, Additional file 1). The molecular mass distribution of the components in *C. taeniatus* venom in relation to their total ion current intensity showed a bimodal distribution*.* The molecular mass can be observed with one major mode (500-3,000 Da) and one minor mode (3,000-7,000 Da). These results clearly show that *C. taeniatus* peptides between 1,000 and 2,000 Da are highly represented compared with those of other molecular masses.

### Conopeptides with disulfide bridges and cysteine distribution

**Figure 4.**
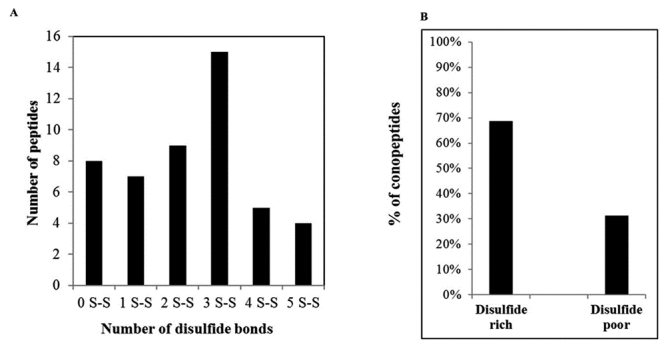
Number of disulfide bridges determined based on the mass shift detected by LC/MS after reduction/alkylation of Cys residues.

LC/MS analysis of the DTT-reduced venom component derivatives of *C. taeniatus* demonstrated an increase in molecular mass by 116.058 × *n* Da. Disulfide bond-containing components were detected in C. *taeniatus* venom (Additional file 2). Forty disulfide bond-containing components were confirmed and the cysteine distribution of those conopeptides is shown in Figure 4A and Additional file 3. The number of disulfide bonds ranged from one to five, and the 0-, 2-, and 3-disulfide frameworks were common in the *C. taeniatus* conopeptides. Peptides contained a 6-cysteine framework, which represents three disulfide bridges, were the most common in the venom. Conopeptides were also divided into “disulfide-poor” (containing two or no cysteines) and “disulfide-rich” (containing four to ten cysteines) groups. The results revealed that 68.75% of the identified peptides were disulfide-rich and the remaining 31.25% were mostly disulfide-poor (Figure 4B and Additional file 3).

### Conotoxin diversity of *C. taeniatus* with respect to superfamily

**Figure 5.**
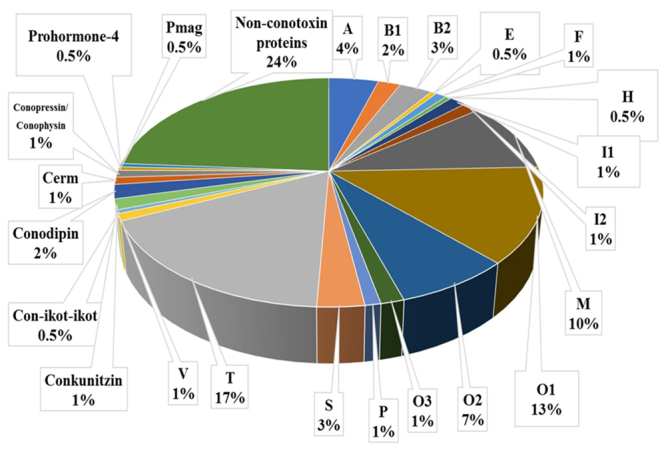
Percentage composition of conotoxin superfamilies and non-conotoxin proteins in *C. taeniatus* venom proteome. The relative abundance of conopeptide superfamilies in *C. taeniatus* venom is expressed as the percent relative abundance of total identified proteins by LC-MS/MS.

A total of 290 peptide fragments (Additional file 4) were detected in the venom of *C. taeniatus*. A protein sequence similarity search in the database revealed that 170 peptides belonging to 153 conotoxin proteins were assigned to 23 conopeptide superfamilies: the A, B1, B2, E, F, H, I1, I2, M, O1, O2, O3, P, S, T, V, Conkunitzin, Con-ikot-ikot, Conodopin, Cerm, Pmag and two hormone families (Conopressin/Conophysin and prohormone-4). The sequences of these peptides are shown in Table 1. Notably, T, O1, M and O2 constitute the highest percentages (22.87%, 17.56%, 13.1% and 9.8%, respectively) of the known superfamilies. Furthermore, some rare superfamilies of conotoxins were found in the venom of *C. taeniatus*. Only one peptide fragment sequence was detected from each of the following conotoxin superfamilies: E, H, Con-ikot-ikot, Pmag and Prohormone-4 (Figure 5). Additionally, 48 non-conotoxin proteins were identified including conoporin, protein disulfide isomerase, arginine kinase and Kazal proteinase inhibitor (Table 2).

**Table 1.** List of identified conotoxin proteins in the venom of Egyptian *C. taeniatus* with their corresponding gene superfamilies, type of targets and possible applications*.*

Protein superfamily	Sequence	Identified protein	Protein accession no.	No. of proteins	Type of target	Possible application
A	QKGLVPSVITTCCGYDPGTMCPPCR	α-Conotoxin S4.4 [*C. striatus*]	AGK23185.1	9	Neuronal/neuromuscular nicotinic acetylcholine receptors (nAChR), K^+^ channels, Na^+^ channels	Treatment of pain, neuronal disorder diseases such as epilepsy, schizophrenia, nicotinic addiction, Alzheimer’s and Parkinson’s disease
	KTAQDNTDLNLITDLNAREDKPK	α-Conotoxin [*C. betulinus*]	AMP44778.1			
	LRECCGRVGPMCPK	α-Conotoxin [*C. bullatus*]	P0CY81.1			
	DERSDMYELKR	α-Conotoxin [*C. achatinus*]	ABD33864.1			
	EVSGSCSSR	α-Conotoxin [*C. stercusmuscarum*]	P0DPM2.1			
	MATLPSCPRHIVR	α-Conotoxin [*C. bullatus*]	P0CY88.1			
	YDKAGNGKYK	α-Conotoxin [*C. flavidus*]	ATF27517.1			
	DMEKKTVEALNTLEGELK	α-Conotoxin [*C. geographus*]	BAO65582.1			
	AAKFKAPALMELTVR	α-Conotoxin [*C. lividus*]	AFD18493.1			
B1	EVAETVRELDAA	Conantokin-Qu [*C. quercinus*]	P0DOZ4.1	4	*N*-methyl-D-aspartate receptor (NMDA) antagonists	Treatment of pain and epilepsy
	SSEEEREHAEKLMTFQNQR					
	SSARSTDDNGNDR	Conantokin-R [*C. radiatus*]	P58806.2			
	AMAELEAKKAQEALK	Conantokin-Oc [*C. ochroleucus*]	P0DP00.1			
	TFEDVEELGKELDANLTK	Conantokin-L2.2 [*C. literatus*]	ADZ72981.1			
B2	FNEGNKSPFDAEGGFGNFMNFMKENSN	Conotoxin precursor superfamily B2 [*C. ermineus*]	AXL95666.1	6	Unknown	Unknown
	FNEGNKSPFDAEGGFGNFMNFMKEN					
	DNLGGFMNFMK	Conotoxin precursor superfamily B2 [*C. ermineus*]	AXL95405.1			
	RDGAPADTANLQPFNQGMQAMPA	Conotoxin precursor superfamily B2 [*C. betulinus*]	AMP44597.1			
	DGAPADAANLQSFDPGMQAMPGMPNM					
	QHSQFNADENKA	Conotoxin precursor superfamily B2 [*C. magus*]	QFQ60977.1			
	EGAPADAANLQSFDPALMPMQGMQG					
	QMAGKASDQFLPFNPN	Conotoxin precursor superfamily B2 [*C. magus*]	DAC80549.1			
	NFDELVND	Conotoxin precursor superfamily B2 [*C. magus*]	QFQ60978.1			
E	TCVALSSLNECAVREK	Conotoxin precursor superfamily E [*C. ermineus*]	AXL95533.1	1	Unknown	Unknown
F	GQKLMHACSIANKYTYD	Conotoxin precursor superfamily F [*C. magus*]	QFQ60998.1	2	Unknown	Unknown
	LMHACSIANK					
	VYHSMMGDMVTCLNHFFRR					
	MNPYSPMNPVNSLYNPMK	Conotoxin precursor superfamily F [*C. magus*]	QFQ60999.1			
H	SLVYVNLKK	Conotoxin precursor superfamily H [*C. ermineus*]	AXL95408.1	1	Unknown	Unknown
I1	MKLALTFLLILMILPLTTGGK MKLALTFLLILMILPLTTGGKK	Conotoxin Im11.13 [*C. imprialis*]	ADZ74137.1	3	Na^+^ channels activator	Treatment of heart failures and pain
	FQKTVPNKCAGDIEI	Contoxin M11.2 [*C. magus*]	P0C613.1			
	EDSLNCIETMATTATCMKSNK	G115_VD_Superfamily_I1_precursor_conopeptide [*C. geographus*]	BAO65648.1			
I2	LSLASSAVLMLLLLFALGNFVGVQPGQITR	Conotoxin Im9.12 [*C. imprialis*]	ADZ99328.1	3	K^+^ channels	Treatment of neuronal disorder diseases and cancer
	SLNECAVR	Conotoxin Sx11.2 [*C. striolatus*]	P0C258.1			
	NEEDHLRLISMQKGGNLK	Conotoxin Gla-TxX [*C. textile*]	Q5I4E6.1			
M	QDLHPNERTGFILPAMR	MLKM group conopeptide Eb3-H0 [*C. ebraeus*]	AEX60108.1	20	K^+^ channels, Na^+^ channels and nAChR	Useful for treatment of pain, stroke, epilepsy, neuronal disorder diseases and cancer
	QDISPNERKR	MLKM group conopeptide Vr3-DPP03 [*C. varius*]	AEX60203.1			
	YAENKQDLNPAER	MLKM group conopeptide [*C. magus*]	DAC80581.1			
	YGWTCWLGCSPCGC	Mu-conotoxin PnIVB [*C. pennaceus*]	P58927.2			
	LTYHAGCPVLMGNKWIANKWIWHYGNMFR	conotoxin superfamily M [*C. eburneus*]	ACV87167.1			
	KYMYNIQR	conotoxin superfamily M [*C. magus*]	DAC80582.1			
	LATSLGDLR	conotoxin precursor superfamily M [*C. ermineus*]	AXL95471.1			
	QDLNLDERR	MLKM group conopeptide Co3-S01 [*C. coronatus*]	AEX60110.1			
	SLKCCSGR	Conotoxin superfamily M **[** *C. magus*]	QFQ61028.1			
	VDGLNHPEPSFGED	Conomarphin conotoxin precursor analog Bt2 [*C. betulinus*]	AGE10520.1			
	NVENKQDLNLDKR	MLKM group conopeptide Ec3-DA01 [*C. emaciatus*]	AEX60080.1			
	QDLNLDKRR					
	RGIKLLAQR					
	DVKCIGSCDSTVWHRV	MLKM group conopeptide [*C. distans*]	AGE10511.1			
	QDLNPDERMKFK	MLKM group conopeptide Cp3-I02 [*C. capitaneus*]	AEX60051.1			
	MQDDISSEQNPLLEKR	Mu-conotoxin BuIIIB [*C. bullatus*]	C1J5M6.1			
	DQDLVEQYRNLK	conotoxin superfamily M [*C. magus*]	QFQ61035.1			
	RCCRVICSR	Conotoxin TxMMSK-01 [*C. textile*]	Q9BPJ1.1			
	EDGKSAALQPWFD	Conotoxin Lt3.7 [*C. literatus*]	ADZ99311.1			
	TLLRQWNK	Conotoxin Reg3.17 [*C. regius*]	A0A2I6EDN0.1			
	QSVTLSNDNRLTADHPNTFYLLIR	Conotoxin precursor superfamily M [*C. ermineus*]	AXL95450.1			
	SVGSSTADCLDNK	Conotoxin precursor superfamily M [*C. rattus*]	AEX60323.1			
O1	LTCMMIVAVLSLTAWTFATADDPR	Conotoxin precursor superfamily O1 [*C. episcopatus*]	BAS22635.1	27	Ca^+^ channels, K^+^ channels, Na^+^ channels and nAChR	Useful in pain, stroke, hypertension, arrhythmias, epilepsy, neuronal disorder diseases and cancer
	FDNDCCDACMLREKQQPICAV	Conotoxin precursor superfamily O1 [*C. miles*]	Q3YEG3.1			
	TASKLLQGSQVAASPL	Conotoxin precursor superfamily O1 [*C. ermineus*]	AXL95342.1			
	NELENLFPKARHEMD	Conotoxin precursor superfamily O1 [C. ermineus]	AXL95353.1			
	NELESYAYSLKNQVNDKEK					
	KCLGFGEACLMFYSDCCSFCVRAVCL	Conotoxin precursor superfamily O1 [*C. episcopatus*]	BAS22577.1			
	GKGAPCRK	Conotoxin precursor superfamily O1 [*C. magus*]	1OMN_A			
	NGLGNLFSNAHHEMK	Conotoxin precursor superfamily O1 *C. episcopatus*]	BAS22395.1			
	MKNPEASKLNNR	Conotoxin precursor superfamily O1 *C. episcopatus*]	BAS22442.1			
	QVYRAVGLTDKMR	Conotoxin precursor superfamily O1 [*C. magus*]	QFQ61065.1			
	ARNELQKLEASQLNER	Conotoxin precursor superfamily O1 [*C. virgo*]	Q3YED8.1			
	DKQEHPAVRGSDDMQDSEDLK	Conotoxin precursor superfamily O1 [*C. arenatus*]	Q9BP77.1			
	SHNCCGVCMIRKLPK	Conotoxin precursor superfamily O1 [*C. ermineus*]	AXL95510.1			
	ALMSTGTNYRLLK	Conotoxin GeXXXIA [*C. generalis*]	A0A2I4QAG8.1			
	NIDGREASGLRK	Conotoxin precursor superfamily O1 [*C. ermineus*]	AXL95529.1			
	RYYTCVALS	Conotoxin precursor superfamily O1 [*C. episcopatus*]	BAS22584.1			
	YYTCVALS					
	MLSMLAWTLMTAMVVMNA	Conotoxin precursor superfamily O1 [*C. episcopatus*]	BAS22670.1			
	CIVGTPCHVCRSQSKSCNGWLGK	Conotoxin Bu6 [*C. bullatus*]	P0CY65.1			
	LEKRDCQDK	Conotoxin Tx6.6 [*C. textile*]	P0DPM4.1			
	GLGYLTFCPSNLGTTLR	Conotoxin precursor superfamily O1 [*C. episcopatus*]	BAS22548.1			
	LDFGDLDPKNE	Conotoxin precursor superfamily O1 [*C. ermineus*]	AXL95735.1			
	CKSPGTPCSKGMR	Conotoxin precursor superfamily O1 [*C. geographus*]	BAO65621.1			
	VGTGLGEYMFDK	Conotoxin ArMKLT1-02 [*C. arenatus*]	Q9BP99.1			
	MLSMLAWTLMTAMVVMNA	Conotoxin precursor superfamily O1 [*C. episcopatus*]	BAS22670.1			
	LNHPEPDFGDLSKLGFGNLDPGG	Conotoxin precursor superfamily O1 [*C. episcopatus*]	BAS22556.1			
	LSATPGFKD	Conotoxin precursor superfamily O1 [*C. episcopatus*]	BAS22677.1			
	NLLKIGTRGQGGCVPPGGGR	Conotoxin precursor superfamily O1 [*C. geographus*]	BAO65614.1			
	MTKRCMHPEGGCR	Conotoxin AbVIE [*C. abbreviates*]	Q9UA85.1			
O2	KEVGNPKASK	Contoxin TxMEKL-P2 [*C. textile*]	Q9BPA9.1	15	Neuronal pacemaker channels and Ca^+^ channels	Useful in pain, hypertension, arrhythmias, epilepsy
	IMEKLTIMLLVAAILMLT	Contoxin [*C. ermineus*]	AXL95503.1			
	QEDPVVRSSDKVQR	Contoxin Bt6.2 [*C. betulinus*]	AGE10507.1			
	EKLTVLILVATVLLAIQVLVQSDREKPLK	Contoxin Tx15a [*C. textile*]	AGK23206.1			
	EMINVLSKGKTNAER	Contoxin MaI51 [*C. marmoreus*]	QFQ61084.1			
	LIILLLVAAVLMSTQALFQEKRPMKK	Contoxin Vc6.12 [*C. victoriae*]	G1AS78.1			
	EKLTVLILVAIVLLTIQVLGQSDRDK	Contoxin Ml15b [*C. miles*]	C8CK75.1			
	SSVDEKIKNK	Conotoxin precursor superfamily O2 [*C. episcopatus*]	BAS22689.1			
	MLSGNNEKR	Conotoxin precursor superfamily O2 [*C. magus*]	QFQ61085.1			
	MEKLTILLLVAALLVLTQALIQGGVEK	Conotoxin Fla6.7 [*C. flavidus*]	AFU50755.1			
	AEINFLSK	Conotoxin precursor superfamily O2 [*C. terebra*]	AGK23197.1			
	MEKLTILLLVAAVLMSTQALIQEKRPK	Conotoxin VnMEKL-0222 [*C. ventricosus*]	AXL95751.1			
	YYTCVALSSLNECAVR	Conotoxin VnMEKL-0111 [*C. ventricosus*]	Q9BPC4.1			
	AKIDFSNR	Conotoxin Vc6.10 [*C. victoriae*]	G1AS76.1			
	LMSAQALMQEK	Conotoxin precursor superfamily O2 [*C. ermineus*]	AXL95556.1			
O3	AKPEFMAAAAK	Conotoxin precursor superfamily O3 [*C. ermineus*]	AXL95644.1	3	Unknown	Induce sleep in mice
	GEKQAMQR	Conotoxin precursor superfamily O3 [*C. ermineus*]	BAO65632.1			
	EQNKTCCGLTNGRPRCVGVCFG	Conotoxin VnMSGL-0123 [*C. ventricosus*]	Q9BP59.1			
P	LSLASSAVLMLLLLFALGNFVGVQPGQITR	Conotoxin Im9.12 [*C. imprialis*]	ADZ99328.1	2	May target a glycine receptor	Induce hyperactivity and spasticity in mice
	QHSSDAVDLQTGQIK	Conotoxin Fla9.1 [*C. flavidus*]	AFU50766.1			
S	KTHLKSGFYR	Conotoxin Tx8.1 [*C. textile*]	AGK23266.1	6	Serotonin receptor or nAChR	Treatment of pathologies including neuropathic pain.
	CYCKNGGR	Conotoxin precursor superfamily S [*C. ermineus*]	AXL95481.1			
	YDNNLCGK	Conotoxin precursor superfamily S [*C. ermineus*]	BAS22723.1			
	QLKCHRNFSVDK	Conotoxin precursor superfamily S [*C. ermineus*]	BAS22797.1			
	CFGESNCR	Conotoxin precursor superfamily S [*C. ermineus*]	BAS22751.1			
	NKIQRSDYLK	Conotoxin precursor superfamily S [*C. ermineus*]	BAS22859.1			
T	DDMSPASFHDNAKRTQHVFWSK	Conotoxin precursor superfamily T [*C. episcopatus*]	BAS25056.1	35	Noradrenaline transporter, somatostatin-3 receptor and possibly Ca^2+^ channels and Na^+^ channels.	Treatment of pain, stroke, hypertension, arrhythmias, epilepsy
	CLPVLIILLLLTASGPSIEARPR	Conotoxin precursor superfamily T [*C. episcopatus*]	BAS25321.1			
	ETDKNLDAVR	Conotoxin Ts5.7 [*C. tessulatus*]	AGK23242.1			
	ECCSDGWCCPQNLK	Conotoxin precursor superfamily T [*C. episcopatus*]	BAS23229.1			
	RCLPVFVILLLLIAFAPSVDVRPKAK	Conotoxin precursor superfamily T [*C. episcopatus*]	BAS23428.1			
	RCLPVLVILLLLIA**S**APSVDVRPKAK	Conotoxin precursor superfamily T [*C. episcopatus*]	BAS25341.1			
	DDMPLASFHANVK	Conotoxin Mr5.2 [C. marmoreus]	Q6PN84.1			
	IQMEKTTVDALNTL	Conotoxin precursor superfamily T [*C*. episcopatus]	BAS25067.1			
	MRCLPVFVILLLLIASAPSVDVLLKAK	Conotoxin precursor superfamily T [*C. episcopatus*]	BAS23994.1			
	TLQTPLNK	Conotoxin precursor superfamily T [*C. episcopatus*]	BAS24922.1			
	LCLPVFIILLLLVSPAATLRVQSKLER	Conotoxin Qc5.4 [*C. quercinus*]	AGK23244.1			
	MRCLPVLIILLLLTASGPSVDAKVHLK	Conotoxin precursor superfamily T [*C. episcopatus*]	BAS25470.1			
	EPYFGEDKLDFGDLDPK	Conotoxin precursor superfamily T [*C. episcopatus*]	BAS24907.1			
	CLPVFVILLLLIASTPNVDALPKTK	Conotoxin precursor superfamily T [*C. episcopatus*]	BAS24300.1			
	FGYRNMTLDETPAKCPWM	Conotoxin precursor superfamily T [*C. episcopatus*]	BAS24141.1			
	DDVPLASFHEDANGILQMLWK	Conotoxin Pu5.1 [C. pulicarius]	BAS24327.1			
	NLQTLLNK	Conotoxin precursor superfamily T [*C. episcopatus*]	BAS24637.1			
	LCLPVFIIPLLLVSPAATLRVQSKLER	Conotoxin Lv5.5 [*C. lividus*]	AGK23255.1			
	CGKNCCPKGWGCIR	Conotoxin Im5.1 [*C. imprialis*]	Q9U6Z5.1			
	MRCLPVFVILLLLIASTPIVDALLKTK	Conotoxin precursor superfamily T [*C. episcopatus*]	BAS24288.1			
	CSEIKENDFG	Conotoxin precursor superfamily T [*C. episcopatus*]	BAS24381.1			
	CLPVFIILLLLIPSALSLIAKPK	Conotoxin precursor superfamily T [*C. magus*	QFQ61106.1			
	QKTKDDIPQASFQDNAK	Conotoxin precursor superfamily T [*C. episcopatus*]	BAS23977.1			
	QLSVELDLQR	Conotoxin Vx5.2 [*C. vexillum*]	AGK23237.1			
	RILQVLENK	Conotoxin precursor superfamily T [*C. episcopatus*]	BAS23633.1			
	GGGPLSSFRDNAK	Conotoxin precursor superfamily T [*C. episcopatus*]	BAS25155.1			
	KGVEAVIK	Conotoxin Ts5.5 [*C. tessulatus*]	Q9BP46.1			
	RCLPVFVILLLLIASAPSVDALPR	Conotoxin precursor superfamily T [*C. episcopatus*]	BAS23533.1			
	CFPVFVILLLLIATAPSVDVRPKAK	Conotoxin precursor superfamily T [*C. episcopatus*]	BAS23041.1			
	MGEVPLNTCPEL	Conotoxin precursor superfamily T [*C. episcopatus*]	BAS24903.1			
	DMPLASSQANVK	Conotoxin mr5.3 [*C. marmoreus*]	Q6PN83.1			
	MTDSYTGENECFYDNNLCGK	Conotoxin precursor superfamily T [*C. episcopatus*]	BAS25019.1			
	TGENECFYDNNLCGK					
	YTGENECFYDNNLCGK					
	KPAQFNSPQEVKEYVRK	Conotoxin precursor superfamily T [*C. ermineus*]	BAS23732.1			
	TVPDDVNAER	Conotoxin Ts5.5 [*C. tessulatus*]	Q9BPF7.1			
	ESKAKLDSLGR	Conotoxin precursor superfamily T [*C. episcopatus*]	AXL95513.1			
V	SDPPVSLVKVDCTAETK	Conotoxin Vi15a [*C. virgo*]	B3FIA5.1	2	Unknown	Unknown
	LGLTEFEAIQEMR	Conotoxin Fla15.3 [*C. flavidus*]	AFU50801.1			
Con-ikot-ikot	SDVERALNIEIRR	Con-ikot-ikot [*C. magus*]	QFQ60982.1	1	α-amino-3-hydroxy-5-methyl-4-isoxazole propionic acid (AMPA) receptors	Inhibiting channel desensitization
Conkunitzin	LEPDAGLCR	Conkunitzin [*C. ermineus*]	AXL95648.1	3	K^+^ channels	Neuronal disorder diseases and cancer
	ISMQKGGNLK	Conkunitzin [*C. magus*]	DAC80559.1			
	SMQKGGNLK					
	RMGEVPLNTCPELFE	Conkunitzin [*C. ermineus*]	AXL95589.1			
Conodipin	HFLAACDR	Conodipin [*C. magus*]	DAC80618.1	4	Conotoxin with PLA_2_ activity.	Potent neurotoxicity, Neurologic application for pain reduction
	QVASDRATSIAR	Conodipin [*C. purpurascens*]	QEO32927.1			
	LISMQMGGNLK	Conodipin [*C. buxeus loroisii*]	ATJ04131.1			
	ACFIRNCPK	Conodipin [*C. ermineus*]	AXL95508.1			
Cerm	CSTDSDHTITVVQSYINGYPEKR	Cerm-13 [*Pionoconus magus*]	QFQ61140.1	2	Unknown	Unknown
	QVCPTMTDSYTGENECFYDNNLCGK					
	CVALSSLNECAVR					
	CSSRCYCKNGGR					
	LTPDKVEMATLTR	Cerm-18 [*C. ermineus*]	AXL95455.1			
Conopressin/Conophysin	RATKECMYCSLGQCVGPR	Conopressin/ Conophysin [*C. ermineus*]	AXL95508.1	2	Vasopressin receptors	Antidiuresis, stimulation of liver glycogenolysis, and central regulation of somatic functions
	ACFIRNCPK					
	DPISVKVLCR	Conopressin/Conophysin [*C. magus*]	DAC80606.1			
Prohrmone-4	YALRLATSLGDLRWSLALTDENINNTK	hormone superfamily prohormone-4	DAC80609.1	1	Unknown	Unknown
Pmag	QVALGLEEGWR	Pmag295 ferritin [*C. magus*]	AXL95451.1	1	Iron receptor	Unknown
**Total**	**170**			**153**		

**Table 2:** List of non-conotoxin proteins identified in Egyptian *C. taeniatus* venom by using MALDI/TOF/MS*.*

Protein family	Sequence	Identified protein	Protein accession no.	No. of proteins	Biological process
Conoporin	VSCIIQVENWTR	Conoporin [*C. lividus*]	ATG85040.1	8	Punching Holes in Membranes. Osmotic stress and cell death of microorganisms
	LVASEVVTPG				
	AEGAMTNGNHAQVK	Conoporin [*C. ebraeus*]	ASF90529.1		
	VIVRPTRNNWK				
	YSNWMGLGMTR				
	VQVENWTRYPLMTPR				
	GKREAFAVR	Conoporin [*C. magus*]	DAC80623.1		
	LQTIYAKDK	Conoporin [*C. consors*]	P0DKQ8.1		
	EAFAVQMPSSGR	Conoporin [*C. ermineus*]	AXL95502.1		
	RFVLMWSAPFDFN	Conoporin [*C. lividus*]	ATG85040.1		
	LGLTEFEAIQEMR	Conoporin [*C. monile*]	ANC48005.1		
	ALQQKRSLQR	Conoporin [*C. magus*]	QFQ61164.1		
Protein disulfide isomerase	TFIDSDEVIVMGFFKDQEGKGA	Protein disulfide isomerase [*C. ebraeus*]	ASF90532.1	27	Oxidative folding of conopeptides
	VLFIYLDTAKT				
	EDVVFGITSEDSVFKEHK	Protein disulfide isomerase [*C.geographus*]	AMM62652.1		
	GKVLFIYLDTAKEENEHI	Protein disulfide isomerase [*C. ermineus*]	AXL95726.1		
	ADSPAMRLIQLGEDLAK	Protein disulfide isomerase [*C. magus*]	QFQ61177.1		
	LFIYLDTAKEESEHIMGFFGLKAADAPTMR	Protein disulfide isomerase [*C. lividus*]	ATG85035.1		
	TENFDKFIK				
	FFMNGQSVDYTGGRQ	Protein disulfide isomerase [*C. bullatus*]	AMM62658.1		
	GSNIKLAKVDATVEK	Protein disulfide isomerase [*C. ermineus*]	AXL95393.1		
	LAKVDIIAEMD	Protein disulfide isomerase [*C.geographus*]	AMM62650.1		
	LAKVDIIAEM				
	QLAPQYSAAA	Protein disulfide isomerase [*C. literatus*]	ARS01447.1		
	TVETDLAGKFEVK	Protein disulfide isomerase [*C. magus*]	DAC80628.1		
	TAQKIFAGDIQNH				
Protein disulfide isomerase	EDWDAQPVKVLVR	Protein disulfide isomerase [*C. frigidus*]	ARU12136.1	27	Oxidative folding of conopeptides
	EGAEDILDTF				
	QLAPQYSAAAG	Protein disulfide isomerase [*C. literatus*]	ARS01447.1		
	DATIEKDLAGK				
	AVLNGEVEAYLK	Protein disulfide isomerase [*C. magus*]	QFQ61181.1		
	QTSDFITWLKKK	Protein disulfide isomerase [*C. monile*]	ANC47993.1		
	MDSMANELEEIQ	Protein disulfide isomerase [*C.geographus*]	AMM62651.1		
	APMYSKAAGK	Protein disulfide isomerase [*C. magus*]	QFQ61177.1		
	DLASKFEVKGFPT	Protein disulfide isomerase [*C. bullatus*]	AMM62660.1		
	GITSEDSVFEEHKMK	Protein disulfide isomerase [*C. magus*]	DAC80628.1		
	STMTKFVQDF	Protein disulfide isomerase [*C. araneosus*]	AQM52452.1		
	DTPAMRLIQLGK				
	KAADTPAMRLIQLGK				
	YKPESDSLDKSTMTKF				
	VAAEIDNIAFGI				
	VQNYLMLFVK	Protein disulfide isomerase [*C.geographus*]	AMM62653.1		
	ITSEDSIFK	Protein disulfide isomerase [*C. ebraeus*]	ASF90532.1		
	NDFSGDFEEAAMSKFVKD	Protein disulfide isomerase [*C. ermineus*]	AXL95421.1		
	GKLMDEGSSIK	Protein disulfide isomerase [*C. miles*]	AQQ10870.1		
	KLATVFSLTLLAFVACEEVKQEEK				
	FIKDNYLPLINEFTQETSQKL	Protein disulfide isomerase [*C. ermineus*]	AXL95599.1		
	TCDQAKTFIDSDEVIVMGFFKDQEGK	Protein disulfide isomerase [*C. textile*]	AMM62657.1		
	TGDVQSYLMLFIK	Protein disulfide isomerase [*C. bullatus*]	AMM62659.1		
	DLAGKFNVTSYPTIKF				
	VEYKGEQK	Protein disulfide isomerase [*C. magus*]	DAC80629.1		
Arginine kinase	LAATPEFK	Arginine kinase [*C. anemone novaehollandiae*]	ADK73590.1	3	Neurotoxicity, leading to paralysis and subsequent death of prey
	KGVEAVIK	Arginine kinase [*C. ebraeus*]	ASF90538.1		
	IQMEKTTVDALNTLEGELAGTYYPLLG	Arginine kinase [*C. miles*]	AQQ10876.1		
ATP synthase F0 subunit 8	VMFSGKGLDCADLK	ATP synthase F0 subunit 8 [*C. betulinus*]	YP_009538431.1	3	important enzyme that provides energy to be used by the cell through the synthesis of ATP
	IMFSSKSLTYINLGKENK	ATP synthase F0 subunit 8 [*C. iosephinae*]	ATZ70391.1		
	KIMFSSKSSTYTNLSK	ATP synthase F0 subunit 8 [*C. borgesi*]	YP_003204749.1		
Kazal protease inhibitor	CAGDIEICK	Kazal protease inhibitor [*C. ermineus*]	AXL95542.1	1	Inhibits serine proteases, including trypsin, chymotrypsin, and elastase.
NADH dehydrogenase subunits	NNSSEVDIIMSK	NADH dehydrogenase subunits [*C. pseudonivifer*]	ATZ70266.1	6	Mitochondrial membrane respiratory
	ELFVLFCVMSGGSALIGGMGGLNQTQVR	NADH dehydrogenase subunits [*C. (Lautoconus) sp*]	ATZ70968.1		
	SIVVMISMLNVVGSVLILLSNFAEGM	NADH dehydrogenase subunits [*C. geographus*]	ATZ70864.1		
	LGILLANFLILVIFPLAGK	NADH dehydrogenase subunits [*C. venulatus*]	APH08616.1		
	MLGILLANFLILVILPLISKKWSWYLK	NADH dehydrogenase subunits [*C. trochulus*]	ATZ69913.1		
	ETSKPASQLILN	NADH dehydrogenase subunits [*C. borgesi*]	YP_003204755.1		
**Total**	**64**			**48**	

## Discussion

The venom components of marine cone snails have evolved bioactive peptides targeting various biological activities to quickly paralyze their preferred prey. Studies have focused on both fish- and mollusk-hunting cone snail venoms because of the biomedical interest of their conopeptides [23]. Information on the peptide profile of worm-hunting species remains limited, despite their significance as a source of pharmacological compounds [24-26]. Thus, vermivore snails might also be promising pharmacological sources [27,28].

It is technically difficult to determine the precise number of components in the venom using biological activity methods [29]. In contrast, LC/MS supplied with an ESI source (LCMS-IT-TOF) is an effective way to provide an abundance of valuable data. This approach revealed a high degree of conopeptide diversity and increased the predicted number from 200 to >1100 distinct toxins per *Conus* species. In the present study, we observed diverse components in the venom of *C. taeniatus*. After mass deconvolution and filtering, a total of more than one hundred different molecular masses were detected from the venom of *C. taeniatus*. Previous studies reported between 50 and 1,000 conopeptides for a *Conus* species [14,30,31]. This variability may enable *C. taeniatus* to modify the composition of the injected venom according to the predatory or defensive stimuli. A total of 276, 298 and 488 different molecular masses were identified in *C. imperialis, C. fulgetrum* and *C. crotchii* venoms, respectively [14,32]. Furthermore, more than 500 different compounds were detected in the venom of *C. consors* by MALDI-MS alone and more than 700 by ESI-MS [33]. In our proteomic study, LCMS-IT-TOF and MS/MS were used to discover the peptide profile and predict putative conotoxin gene superfamilies in the neglected worm-hunting snail *C. taeniatus.* The number of distinct peptides previously reported in different species varies considerably. For example, 290 peptides were detected in *C. taeniatus* venom (this study), 1,746 peptides in the venom of *C. textile* [14], and 8,000 peptides in the venom of *C. marmoreus* [34]. Significant differences in peptide numbers in the proteomic analysis of *Conus* species may be due to the difference in methods of venom collection, total number of collected specimens and pooled data, or different conditions used for peptide authentication [35,36].

In the present study, we reported that the majority (84%) of *C. taeniatus* components were 500-4,000 Da, whereas only 16% of all components were large peptides (>4,000 Da). In addition, over 50% of the conopeptides detected in the venom of the studied species were smaller than 2,500 Da. [37]. Similarly, low molecular weight peptides were the most abundant in *C. fulgetrum* venom [37], *C. marmoreus* and *C. bandanus* venoms [2]. Although these species share worm-like prey, they evolved different strategies to produce diverse conopeptides. Low molecular weight peptides in venom specifically alter Na^+^, Ca^2+^, K^+^, and Cl^-^ ion channels [38,39]. Because these low molecular weight peptides have the ability to block voltage-gated channels, they can be employed in tumor growth impairment [40,41]. Therefore, the discovered low molecular weight peptides in *C. taeniatus* and other *Conus* venoms could be employed in tumor treatment because they can most likely control the signal transduction pathways in malignant tumor cells.

Peptide toxins are usually highly bridged proteins with multiple pairs of intrachain disulfide bonds. The analysis of disulfide connectivity is important in protein structure determination [42]. The disulfide pattern in the venom peptides of *C. taeniatus* was estimated directly by LCMS-IT-TOF without venom fractionation. We reported herein that most *C. taeniatus* peptides were disulfide-rich, with the highest possibility of 3 disulfide bridges. Disulfide-rich peptides were also abundant in the venom of *C. consors* [43], *C. bandanus* and *C. marmoreus* [2] and *C. fulgetrum* [37]. It is well known that disulfide bonds confer conformational stability to folded proteins [44]. Therefore, an understanding of disulfide linkage patterns is necessary for further studies relating the structure to the function of *Conus* venom peptides.

Classical peptide identification methods, including Sanger sequencing and isolation, are generally considered laborious with limited efficiency and are sometimes limited by sample availability. The advance of high-throughput sequencing combined with bioinformatics analysis has allowed for more precise identification of conopeptides to predict and discover novel conotoxins from a variety of *Conus* species [34,45-49]. Here, the majority of conotoxins identified in *C. taeniatus* belonged to the T-superfamily, suggesting an important function for *C. taeniatus*. The T-superfamily peptides in *Conus* venom target different types of ion channels or neurotransmitters [50,51]. Similarly, the T-superfamily is predominant in *C. victoriae* venom [52]. Evidently, the T-superfamily is abundant in *C. taeniatus* and other *Conus* species; however, little is known about this group of conotoxins. Variations in conotoxin targets enable them to be included in the treatment of several diseases, such as pain, cancers and depression [1,53,54]. For example, M-superfamily peptides, which are ubiquitous in *Conus* venom [55], are blockers of voltage-gated sodium and potassium channels or nicotinic acetylcholine receptors. Conopeptides from the O-superfamily, which have O1, O2, and O3 variations, can block voltage-gated calcium and potassium channels [56,57]. Currently, ziconotide from the O1 superfamily is commercially available and works as an analgesic that relieves pain by selectively inhibiting the N-type voltage-gated Ca^++^ channel, and thus inhibiting the release of pro-nociceptive neurochemicals in the spinal cord [58,59]. The M- and O-superfamilies are the predominant superfamilies in *C. tribblei*, *C. bullatus*, *C. marmoreus*, and *C. pulicarius* [52]. Additionally, A-superfamily conopeptides are the most abundant in *C. consors*, *C. geographus*, and *C. bullatus* [52], and together with the O-superfamilies, can block potassium channels and affect nicotinic acetylcholine receptors [32,60]. As conopeptides in *C. taeniatus* can target different ion channels and receptors, they are promising candidate compounds for biomedical applications and drug development.

In addition to conopeptides, different non-conopeptide proteins and enzymes were detected. Conoporin, which is known as a potent cytolytic and hemolytic protein, was detected in *C. taeniatus venom*. Conoporins exert toxicity by forming pores in membranes, leading to cell death [61]. Interestingly, different peptide fragments of conoporins were identified, indicating the potential antimicrobial activity of *C. taeniatus* venom. The enzyme family protein disulfide-isomerase (PDI) was detected in the venom of *C. taniatus* and can catalyze the oxidation, isomerization, and reduction of disulfide bonds to ensure the proper folding of proteins. PDI confers stability to proteins by covalently linking specific cysteine residues [53,62]. This enzyme family has also been identified in the venom glands of several insects, including *Aphidius ervi* [63] and*Psytallia*species [64], and in the crude venom extract of*Pteromalus puparum* [65],*Diversinervus elegans* [66] and*Cotesia chilonis* [67]. In venomous cone snails, PDIs are only located in the venom glands directing the folding of conotoxins but not in the secreted venom [68,69]. PDIs rarely exist in the extracellular space and are principally localized in the endoplasmic reticulum [70]. Therefore, the presence of PDI in the extracted venom of*C. taeniatus* is probably due to the rupture of venom-producing cells during venom collection. In this study, several of the detected protein fragments could not be attributed to conopeptides. One possible explanation is that the extracted venom may contain other untreated peptides and cellular debris. In addition, whole conotoxin sequences are not described and available in the database.

## Conclusion

The data described herein contribute to addressing the gap of knowledge regarding the venom composition of the neglected vermivore cone snail *C. taeniatus* at the proteomic level. We used different proteomic approaches to characterize various peptide compositions of *C. taeniatus* venom. We successfully identified 170 out of 234 peptide fragments and classified them into 23 known gene superfamilies. Many conopeptide superfamilies targeting various types of ion channels and receptors were identified in the venom composition of the worm-hunting *C. taeniatus,* making them valuable lead compounds for drug development and biomedical applications. Therefore, further research with more sensitive methods are required to determine the peptide composition of untapped cone snail venoms.

### Abbreviations

AA: acetic acid; DTT: dithiothreitol; ESI: electrospray ion source; FA: formic acid; HPLC: high-performance liquid chromatography; MALDI-TOF matrix-assisted laser desorption/ionization time-of-flight; MM: Monoisotopic molecular masses; MS: mass spectrometry; PDI: disulfide-isomerase; PTM: posttranslational modifications; RP-HPLC: reversd-phase HPLC. 

## Supplementary material

The following online material is available for this article:

Additional file 1. Monoisotopic molecular masses (MM) of native components in *Conus taeniatus* venom detected by LC/MS analysis.

Additional file 2. Monoisotopic molecular masses (MM) of reduced and alkylated components in *Conus taeniatus* venom detected by LC/MS analysis.

Additional file 3. Estimation of the number of disulfide bridges included in each component in *Conus taeniatus* venom.

Additional file 4. List of peptide sequences detected in *C. taeniatus* venom by using MALDI/TOF/MS.
