# Supplementary material for: Proteomic analysis of Red Sea Conus taeniatus venom reveals potential biological applications
Source: J Venom Anim Toxins Incl Trop Dis. 2021 Oct 18;27:e20210023. doi: 10.1590/1678-9199-JVATITD-2021-0023 (PMC8525892; doi:10.1590/1678-9199-JVATITD-2021-0023)
Supplement: Additional file 1. [file 1678-9199-jvatitd-27-e20210023-s1.pdf]

J Venom Anim Toxins incl Trop Dis
https://doi.org/10.1590/1678-9199-JVATITD-2021-0023

# Supplementary Material to "Proteomic analysis of Red Sea *Conus taeniatus* venom reveals potential biological applications"

**Additional file 1.** Monoisotopic molecular masses (MM) of native components in *Conus taeniatus* venom detected by LC/MS analysis.

| Retention time (min) | Monoisotopic molecular mass (Da) |
|---|---|
| 2.4-3.2 | 536.16 558.14 429.15 840.8 841.3 3046.17 |
| 19.6-20.3 | 1448.5 1470.58 1117.53 |
| 20.3-20.8 | 812.36 |
| 21-21.4 | 1015.47 |
| 22.7-23.6 | 1050.37 1186.56 |
| 23.7-24.2 | 3294.3 3606.44 |
| 24.2-25 | 3291.57 |
| 25.2-25.9 | 3174.22 1815.78 4545.8 4584.71 3388.2 2071.07 1425.6 |
| 25.9-26.4 | 1035.38 1012.5 1162.25 3091.25 1686.73 |
| 26.6-27.4 | 501.22 1002.45 |
| 27.4-28.3 | 3129.14 3315.3 1060.46 |
| 28.3-28.7 | 5758.21 1938.06 2927.01 |
| 28.7-29 | 946.41 2511.98 |
| 29.1-29.4 | 2970.97 2660.98 5325.98 524.28 2952.1 1476.1 2991.93 |
| 29.4-30.1 | 1674.58 1316.45 |
| 30.3-31 | 1235.46 1218.44 3074.1 3003.98 2135.79 3112.06 3098.03 |
| 31-31.3 | 2043.68 3058.01 3133.11 |
| 31.7-32.2 | 1236.45 2913.31 2266.01 1580.75 618.22 2125.08 1973.05 |
| 32.2-33.1 | 963.34 1001.28 981.34 2314.05 944.26 2594.09 2221.01 1793.6 |
| 33.1-33.7 | 3153.01 3211.03 |
| 34.2-34.7 | 4786.9 3195.72 |
| 34.7-35.3 | 4161.67 1923.88 1408.24 2817.16 4221.69 4325.76 1654.62 |
| 37.5-38 | 4308.75 4368.8 2185.39 2184.89 1584.65 |
| 38.3-38.9 | 4282.34 7006.5 6230.14 4281.25 4610.53 1851.65 7782.19 7013.9 |
| 39.1-39.4 | 2397.34 3383.45 |
| 39.4-40 | 4471.01 |
| 40-40.3 | 2355.16 4028.59 |
| 40.3-40.7 | 4188.3 7168.24 11087.19 11082.15 |
| 41.2-41.7 | 700.27 2269.26 1914.1 818.3 1854.89 3259.72 1135.12 1134.62 1636.6 2978.48 2119.1 1400.54 |
| 41.7-42.4 | 1775.6 591.87 1813.53 1799.55 887.8 888.3 1527.45 2006.03 |
| 44.2-45.3 | 1862.61 895.31 894.81 1805.61 1789.61 1900.54 609.18 1886.56 1586.63 901.87 1827.56 1843.56 1683.68 |
| 46.1-46.3 | 1735.7 |
| 46.3-47 | 1270.49 1292.47 1308.43 635.24 |
| 47.1-47.6 | 3424.58 |
| 48.9-49.4 | 12340.22 4995.34 8734.13 7407.48 4367.55 |
| 51-51.4 | 3370.5 |
| 51.8-52.3 | 3408.6 |
| 56.3-56.9 | 2925.3 |
| 58.3-59 | 452.16 436.18 |
| 61.6-62.2 | 3265.32 3288.3 |
