# Supplementary material for: Proteomic analysis of Red Sea Conus taeniatus venom reveals potential biological applications
Source: J Venom Anim Toxins Incl Trop Dis. 2021 Oct 18;27:e20210023. doi: 10.1590/1678-9199-JVATITD-2021-0023 (PMC8525892; doi:10.1590/1678-9199-JVATITD-2021-0023)
Supplement: Additional file 2. [file 1678-9199-jvatitd-27-e20210023-s2.pdf]

J Venom Anim Toxins incl Trop Dis
https://doi.org/10.1590/1678-9199-JVATITD-2021-0023

Supplementary Material to "Proteomic analysis of Red Sea *Conus taeniatus* venom reveals potential biological applications"

Additional file 2. Monoisotopic molecular masses (MM) of reduced and alkylated components in the *Conus taeniatus* venom detected by LC/MS analysis.

Retention time (min)	Monoisotopic molecular mass (Da)						
1.2-1.7	537.07	507.13	470.06	640.15			
4.5-7.5	442.03	410.04	603.08	619.05			
13-14.2	559.05						
16.5-18.2	470.06	486.03					
19.2-19.8	546.06	502.04					
20.5-21	1282.48						
21.8-22.6	484.08	3554.35	413.04				
23.5-24.4	1118.5	2022.74					
25-25.5	498.09	528.05					
25.6-26.4	574.09	1660.08	3510.38	1062.46	1659.57		
26.4-26.9	3522.37						
26.9-27.5	3530.43						
27.5-27.9	5011.01	5009.99	3506.37	3673.37			
28.3-29	3676.01	1467.58	2391.84	3009.18	733.79	734.29	4647
29.2-29.8	1506.49	3642.44	3713.32	1468.58	2863.2		
29.9-30.3	3452.31	3714.33	3486.06	3472.08	3460.17		
30.3-30.6	3422.27	568.3	3446.22	1730.6	1723.62		
30.6-31	4641.91						
31-31.4	1296.73	1703.12	2704.05	3406.26			
31.4-31.9	646.06 2913.27 954.4 1648.56						
32-32.7	3504.13 6128.74 4625.88 4664.84 7000.36 5256.2 7301.22						
33-33.9	2123.77 2161.71 1073.86 2126.37 2147.71						
34.2-34.7	1079.39 1117.33 450.16						
35-35.4	2153.78						
35.4-35.6	2137.8 4772.96 701.76 2175.72 2078.75 2160.79						
35.6-36.2	2159.76 2191.72						
36.3-36.9	2210.77 2248.72 2234.74 1620.63 2233.76						
37.2-37.8	1502.6 1540.54 1526.56 1524.59 770.73 2092.76						
38.4-38.8	7788.76 5404.62						
38.9-39.4	422.09 2195.77 2268.77 1915.78 1818.73						
39.4-40.5	2511.39 2414.29 1616.67						
40.6-41.2	2119.11 2983.34						
41.2-41.5	1914.1 2269.24 1854.89						
42.4-42.6	2695.5 1605.6 2006.01						
50.9-51.4	8967.04 5124.46 6433.66						
58.4-59	452.16 436.18						
