# Supplementary material for: Proteomic analysis of Red Sea Conus taeniatus venom reveals potential biological applications
Source: J Venom Anim Toxins Incl Trop Dis. 2021 Oct 18;27:e20210023. doi: 10.1590/1678-9199-JVATITD-2021-0023 (PMC8525892; doi:10.1590/1678-9199-JVATITD-2021-0023)
Supplement: Additional file 3. [file 1678-9199-jvatitd-27-e20210023-s3.pdf]

J Venom Anim Toxins incl Trop Dis
https://doi.org/10.1590/1678-9199-JVATITD-2021-0023

# Supplementary Material to "Proteomic analysis of Red Sea *Conus taeniatus* venom reveals potential biological applications"

**Additional file 3.** Estimation of the number of disulfide bridges included in each component in the *Conus taeniatus* venom.

| MM native | MM alkylated* | | Mass difference | No. of S-S bond | Difference from calculated value |
|---|---|---|---|---|---|
| 618.22 | 734.28 | (28.3) | 116.06 | 1 | 0.002 |
| 944.26 | 1524.59 | (37.2) | 580.33 | 5 | 0.04 |
| 946.41 | 1062.46 | (26.1) | 116.05 | 1 | -0.008 |
| 946.41 | 1526.56 | (37.3) | 580.15 | 5 | -0.14 |
| 963.34 | 1079.39 | (34.6) | 116.05 | 1 | -0.008 |
| 1001.28 | 1117.33 | (34.7) | 116.05 | 1 | -0.008 |
| 1002.45 | 1118.5 | (23.5) | 116.05 | 1 | -0.008 |
| 1050.37 | 1282.48 | (20.5) | 232.11 | 2 | -0.006 |
| 1060.46 | 1524.59 | (37.2) | 464.13 | 4 | -0.102 |
| 1235.46 | 1467.58 | (28.3) | 232.12 | 2 | 0.004 |
| 1236.45 | 1468.58 | (29.8) | 232.13 | 2 | 0.014 |
| 1270.49 | 1502.6 | (37.2) | 232.11 | 2 | -0.006 |
| 1292.47 | 1524.59 | (37.2) | 232.12 | 2 | 0.004 |
| 1308.43 | 1540.54 | (37.7) | 232.11 | 2 | -0.006 |
| 1586.63 | 1818.73 | (39.4) | 232.1 | 2 | -0.016 |
| 1654.62 | 2234.74 | (36.4) | 580.12 | 5 | -0.17 |
| 1674.58 | 2022.74 | (24.3) | 348.16 | 3 | -0.014 |
| 1683.68 | 1915.78 | (39.4) | 232.1 | 2 | -0.016 |
| 1775.6 | 2123.77 | (33.0) | 348.17 | 3 | -0.004 |
| 1789.61 | 2137.8 | (35.4) | 348.19 | 3 | 0.016 |
| 1799.55 | 2147.71 | (33.9) | 348.16 | 3 | -0.014 |
| 1805.61 | 2153.78 | (35.4) | 348.17 | 3 | -0.004 |
| 1813.53 | 2161.71 | (33.5) | 348.18 | 3 | 0.006 |
| 1827.56 | 2175.72 | (35.6) | 348.16 | 3 | -0.014 |
| 1862.61 | 2210.77 | (36.3) | 348.16 | 3 | -0.014 |
| 1900.54 | 2248.72 | (36.3) | 348.18 | 3 | 0.006 |
| 2043.68 | 2159.76 | (35.6) | 116.08 | 1 | 0.022 |
| 2043.68 | 2391.84 | (28.8) | 348.16 | 3 | -0.014 |
| 3058.01 | 3406.26 | (31.4) | 348.25 | 3 | 0.076 |
| 3058.01 | 3522.37 | (26.4) | 464.36 | 4 | 0.128 |
| 3074.1 | 3422.27 | (30.3) | 348.17 | 3 | -0.004 |
| 3098.03 | 3446.22 | (30.6) | 348.19 | 3 | 0.016 |
| 3133.11 | 3713.32 | (29.8) | 580.21 | 5 | -0.08 |
| 3174.22 | 3406.26 | (31.4) | 232.04 | 2 | -0.076 |
| 3174.22 | 3522.37 | (26.4) | 348.15 | 3 | -0.024 |
| 3294.3 | 3642.44 | (29.2) | 348.14 | 3 | -0.034 |
| 3388.2 | 3504.13 | (32.0) | 115.93 | 1 | -0.128 |
| 4161.67 | 4625.88 | (32.4) | 464.21 | 4 | -0.022 |
| 4308.75 | 4772.96 | (35.4) | 464.21 | 4 | -0.022 |
| 4545.8 | 5009.99 | (27.5) | 464.19 | 4 | -0.042 |

*Values in a parenthesis are HPLC retention time (min).
