# Supplementary material for: Proteomic analysis of Red Sea Conus taeniatus venom reveals potential biological applications
Source: J Venom Anim Toxins Incl Trop Dis. 2021 Oct 18;27:e20210023. doi: 10.1590/1678-9199-JVATITD-2021-0023 (PMC8525892; doi:10.1590/1678-9199-JVATITD-2021-0023)
Supplement: Additional file 4. [file 1678-9199-jvatitd-27-e20210023-s4.pdf]

J Venom Anim Toxins incl Trop Dis
https://doi.org/10.1590/1678-9199-JVATITD-2021-0023

Supplementary Material to "Proteomic analysis of Red Sea *Conus taeniatus* venom reveals potential biological applications"

Additional file 4. List of peptide sequences detected in *Conus taeniatus* venom by using MALDI/TOF/MS.

No.	Sequence
1	EGAPADAANLQSFDPALMSMQGMQ
2	YKPESDSLDKSTMTKF
3	GLGYLTFCPSNLGTTLR
4	SHNCCGVCMIRKLPK
5	MTDSYTGENECFYDNNLCGK
6	NIDGREASGLRK
7	VSCIIQVENWTR
8	DPISVKVLCR
9	NVENKQDLNLDKR
10	ETDKNLDAVR
11	EVAETVRELDAA
12	CSTDSDHTITVVQSY
13	ALMSTGTNYRLLK
14	DGAPADAANLQSFDPGMQAMPGMPNM
15	DMEKKTVEALNTLEGELK
16	AMAELEAKKAQEALK
17	NVENKQDLNLDK
18	YTGENECFYDNNLCGK
19	TFIDSDEVIVMGFFKDQEGKGA
20	CSTDSDHTITVVQSYINGYPEKR
21	TGENECFYDNNLCGK
22	SYINGYPEK
23	MKLALTFLLILMILPLTTGGK
24	GKVLFIYLDTAKEENEHI
25	LEPDAGLCR
26	GQKLMHACSIANKYTYD
27	ADSPAMRLIQLGEDLAK
28	ESKAKLDSLGR
29	NEEDHLRLISMQKGGNLK

No.	Sequence
30	**SDVERALNIEIRR**
31	**MTKRCMHPEGGCR**
32	**MATLPSCPRHIVR**
33	**LNHPEPDFGDLSKLGFGNLDPGG**
34	**TVPDDVNAER**
35	**EKLTVLILVATVLLAIQVLVQSDREKPLK**
36	**LFIYLDTAKEESEHIMGFFGLKAADAPTMR**
37	**NLQTLLNK**
38	**IMEKLTIMLLVAAILMLT**
39	**FQKTVPNKCAGDIEI**
40	**SMQKGGNLK**
41	**LISMQMGGNLK**
42	**RGIKLLAQR**
43	**QVCPTMTDSYTGENECFYDNNLCGK**
44	**DERSDMYELKR**
45	**LCLPVFIIPLLLVSPAATLRVQSKLER**
46	**MNPYSPMNPVNSLYNPMK**
47	**QVCPTMTDSYTGENECFYDNNLCGK**
48	**CVALSSLNECAVR**
49	**INGYPEKR**
50	**FNEGNKSPFDAEGGFGNFMNFMKEN**
51	**QVCPTMTDSYTGENECFYDNNLCGK**
52	**FQKTVPNKCAGDIEI**
53	**EKLTVLILVAIVLLTIQVLGQSDRDK**
54	**YDKAGNGKYK**
55	**DVKCIGSCDSTVWHRV**
56	**EDGKSAALQPWFD**
57	**CSEIKENDFG**
58	**EQNKTCCGLTNGRPRCVGVCFG**
59	**MQDDISSEQNPLLEKR**
60	**MRCLPVFVILLLLIASTPIVDALLKTK**
61	**MKLALTFLLILMILPLTTGGKK**
62	**LTEFEAIQEMR**
63	**DDMSPASFHDNAKRTQHVFWSK**
64	**MLGILLANFLILVILPLISKKWSWYLK**
65	**CSTDSDHTITVVQSYINGYPEK**

No.	Sequence
66	**CAGDIEICK**
67	**EDVVFGITSEDSVFKEHK**
68	**QVCPTMTDSYTGENECFYDNNLCGK**
69	**CSSRCYCKNGGR**
70	**QDLNPDERMKFK**
71	**LSLASSAVLMLLLLFALGNFVGVQPGQITR**
72	**LSATPGFKD**
73	**CLPVLIILLLLTASGPSIEARPR**
74	**DPISVKVLCR**
75	**RCCRVICSR**
76	**RCLPVFVILLLLIAFAPSVDVRPKAK**
77	**VKQEEKVYVLNDK**
78	**VLFIYLDTAKT**
79	**QHSSDAVDLQTGQIK**
80	**MEKLTILLLVAALLVLTQALIQGGVEK**
81	**LVASEVVTPG**
82	**TAQKIFAGDIQNH**
83	**RCLPVLVILLLLIASAPSVDVRPKAK**
84	**DQDLVEQYRNLK**
85	**DDMPLASFHANVK**
86	**FNEGNKSPFDAEGGFGNFMNFMKENSN**
87	**VMFSGKGLDCADLK**
88	**NELENLFPKARHEMD**
89	**YALRLATSLGDLRWSLALTDENINNTK**
90	**AEGAMTNGNHAQVK**
91	**ELFVLFCVMSGGSALIGGMGGLNQTQVR**
92	**HFLAACDR**
93	**AKPEFMAAAAK**
94	**QKGLVPSVITTCCGYDPGTMCPPCR**
95	**LISMQKGGN**
96	**QDLHPNERTGFILPAMR**
97	**QDLNLDKRR**
98	**SLNECAVR**
99	**QVCPTMTDSYTGENECFYDNNLCGK**
100	**IQMEKTTVDALNTL**
101	**GEKQAMQR**

No.	Sequence
102	EDSLNCIETMATTATCMKSNK
103	QEDPVVRSSDKVQR
104	EVSGSCSSR
105	QDISPNERKR
106	QVASDRATSIAR
107	FFMNGQSVDYTGGRQ
108	DLAGKFNVTSYPTIKF
109	TVETDLAGKFEVK
110	AGDIEICK
111	EDWDAQPVKVLVR
112	KTAQDNTDLNLITDLNAREDKPK
113	TENFDKFIK
114	CSTDSDHTITVVQSY
115	LRECCGRVGPMCPK
116	MKNPEASKLNNR
117	SIVVMISMLNVVGSVLILLSNFAEGM
118	KIMFSSKSSTYTNLSK
119	CSTDSDHTITVVQSYINGYPEKR
120	ISMQKGGNLK
121	MRCLPVFVILLLLIASAPSVDVLLKAK
122	QVYRAVGLTDKMR
123	TLQTPLNK
124	VDGLNHPEPSFGED
125	QLAPQYSAAAG
126	EMINVLSKGKTNAER
127	IQMEKTTVDALNTLEGELAGTYYPLLG
128	CLPVFIILLLLIPSALSLIAKPK
129	KTHLKSGFYR
130	GKREAFAVR
131	ARNELQKLEASQLNER
132	LCLPVFIILLLLVSPAATLRVQSKLER
133	RATKECMYCSLGQCVGPR
134	MGEVPLNTCPELF
135	QVCPTMTDSYTGENECFYDNNLCGK
136	DKQEHPAVRGSDDMQDSEDLK
137	YAENKQDLNPAER

No.	Sequence
138	**SYINGYPEKR**
139	**DNLGGFMNFMK**
140	**AVLNGEVEAYLK**
141	**MRCLPVLIILLLLTASGPSVDAKVHLK**
142	**CYCKNGGR**
143	**LIILLLVAAVLMSTQALFQEKRPMKK**
144	**IMFSSKSLTYINLGKENK**
145	**LTCMMIVAVLSLTAWTFATADDPR**
146	**SGCENPDSGVG**
147	**KPAQFNSPQEVKEYVRK**
148	**LQTIYAKDK**
149	**VIVRPTRNNWK**
150	**FDNDCCDACMLREKQQPICAV**
151	**CSTDSDHTITVVQS**
152	**YYTCVALSSLNECAVR**
153	**GSNIKLAKVDATVEK**
154	**CSTDSDHTITVV**
155	**CLPVFVILLLLIASTPNVDALPKTK**
156	**LAATPEFK**
157	**RDGAPADTANLQPFNQGMQAMPA**
158	**EAFAVQMPSSGR**
159	**LAKVDIIAEMD**
160	**SSLNECAVR**
161	**LTYHAGCPVLMGNKWIANKWIWHYGNMFR**
162	**MLSMLAWTLMTAMVVMNA**
163	**QLAPQYSAAA**
164	**SSVDEKIKNK**
165	**APMYSKAAGK**
166	**LGILLANFLILVIFPLAGK**
167	**AKIDFSNR**
168	**CIVGTPCHVCRSQSKSCNGWLGK**
169	**KYMYNIQR**
170	**RTLQTLLHK**
171	**KAADTPAMRLIQLGK**
172	**LATSLGDLR**
173	**LAKVDIIAEM**

No.	Sequence
174	**TASKLLQGSQVAASPL**
175	**YDNNLCGK**
176	**MLSGNNEKR**
177	**QTSDFITWLKKK**
178	**MLSMLAWTLMTAMVVMNA**
179	**CSTDSDHTITVVQSYINGYPEKR**
180	**QHSQFNADENKA**
181	**EPYFGEDKLDFGDLDPK**
182	**MLGILLANFLILVIFPLAGKKWSWYLK**
183	**MDSMANELEEIQ**
184	**QVALGLEEGWR**
185	**VEYKGEQK**
186	**NLLKIGTRGQGGCVPPGGGR**
187	**GITSEDSVFEEHKMK**
188	**QDLNLDERR**
189	**QDLNLDERR**
190	**SSARSTDDNGNDR**
191	**DLASKFEVKGFPT**
192	**TLLRQWNK**
193	**AEINFLSK**
194	**QVCPTMTDSYTGENECFYDNNLCGK**
195	**MGEVPLNTCPEL**
196	**CSTDSDHTITVVQSYINGYPEKR**
197	**NGLGNLFSNAHHEMK**
198	**STMTKFVQDF**
199	**ITSEDSIFK**
200	**QSVTLSNDNRLTADHPNTFYLLIR**
201	**VGTGLGEYMFDK**
202	**RFVLMWSAPFDFN**
203	**NNSSEVDIIMSK**
204	**VQNYLMLFVK**
205	**DTPAMRLIQLGK**
206	**CSTDSDHTITVVQSYINGYPEK**
207	**DMPLASSQANVK**
208	**ETSKPASQLILN**
209	**LGLTEFEAIQEMR**

No.	Sequence
210	**FGYRNMTLDETPAKCPWM**
211	**DDVPLASFHEDANGILQMLWK**
212	**KGVEAVIK**
213	**CSTDSDHTITVVQSY**
214	**QKTKDDIPQASFQDNAK**
215	**LEKRDCQDK**
216	**ALQQKRSLQR**
217	**TGENECFYDNNLCGK**
218	**NDFSGDFEEAAMSKFVKD**
219	**SSEEEREHAEKLMTFQNQR**
220	**LMHACSIANK**
221	**EGAPADAANLQSFDPALMPMQGMQG**
222	**QLSVELDLQR**
223	**SLNECAVR**
224	**CGKNCCPKGWGCIR**
225	**DATIEKDLAGK**
226	**GKLMDEGSSIK**
227	**RYYTCVALS**
228	**CSTDSDHTITVVQ**
229	**RMGEVPLNTCPELF**
230	**QVCPTMTDSYTGENECFYDNNLCGK**
231	**FIKDNYLPLINEFTQETSQKL**
232	**RILQVLENK**
233	**YTGENECFYDNNLCGK**
234	**YSNWMGLGMTR**
235	**SDPPVSLVKVDCTAETK**
236	**QVCPTMTDSYTGENECFYDNNLCGK**
237	**GKGAPCRK**
238	**QMAGKASDQFLPFNPN**
239	**CAGDIEICK**
240	**YGWTCWLGCSPCGC**
241	**NELESYAYSLKNQVNDKEK**
242	**LTPDKVEMATLTR**
243	**MGEVPLNTCPELFE**
244	**QVCPTMTDSYTGENECFYDNNLCGK**
245	**YYTCVALSSLNECAVR**

No.	Sequence
246	GGGPLSSFRDNAK
247	MEKLTILLLVAAVLMSTQALIQEKRPK
248	LCLPVFIILLLLVSPAATLRVQSK
249	CSTDSDHTITVVQSY
250	CYCKNGGR
251	LMSAQALMQEK
252	TGENECFYDNNLCGK
253	ACFIRNCPK
254	QVCPTMTDSYTGENECFYDNNLCGK
255	QLKCHRNFSVDK
256	ECCSDGWCCPQNLK
257	AAKFKAPALMELTVR
258	CLPVFVILLLLIASTPSVAALLKTK
259	KCLGFGEACLMFYSDCCSFCVRAVCL
260	SLKCCSGR
261	CSTDSDHTITVVQ
262	YTGENECFYDNNLCGK
263	TCDQAKTFIDSDEVIVMGFFKDQEGK
264	LMHACSIANK
265	RCLPVFVILLLLIASAPSVDALPR
266	VAAEIDNIAFGI
267	EKLTVLILVATVLLAIQVLVQSDREKPLK
268	YYTCVALS
269	MGEVPLNTCPELFE
270	EGAEDILDTF
271	RMGEVPLNTCPELFE
272	LDFGDLDPKNE
273	NFDELVND
274	TCVALSSLNECAVREK
275	KLATVFSLTLLAFVACEEVKQEEK
276	SLVYVNLKK
277	NKIQRSDYLK
278	VQVENWTRYPLMTPR
279	CFPVFVILLLLIATAPSVDVRPKAK
280	CSTDSDHTITVV
281	KNAGICVKA

No.	Sequence
282	**TFEDVEELGKELDANLTK**
283	**KEVGNPKASK**
284	**VYHSMMGDMVTCLNHFFRR**
285	**CFGESNCR**
286	**SVGSSTADCLDNK**
287	**TGDVQSYLMLFIK**
288	**CKSPGTPCSKGMR**
289	**YDNNLCGK**
290	**NNSSEVDIIMS**
